# Bronchoalveolar Lavage in Immunocompetent Patients with Pneumonia: A Retrospective Cohort Study Shows No Survival Benefit

**DOI:** 10.3390/jcm14248785

**Published:** 2025-12-11

**Authors:** Alon Pomerantz, Adar Yaacov, Adam Goldman, Ofir Deri, Asaf Zlotnik, Eden Lahav, Paz Israel Sailes, Ella Huszti, Liran Levy

**Affiliations:** 1 School of Medicine, Faculty of Medical and Health Sciences, Tel-Aviv University, Tel Aviv 6997801, Israel; 2 Internal Medicine Department, Sheba Medical Center, Tel-Hashomer, Ramat Gan 5266202, Israel; 3 Institute of Pulmonary Medicine, Sheba Medical Center, Tel-Hashomer, Ramat Gan 5266202, Israel; 4 The Helmsley Cancer Center, Shaare Zedek Medical Center, Jerusalem 9103102, Israel; 5 Faculty of Medicine, The Hebrew University of Jerusalem, Jerusalem 9112102, Israel; 6 Department of Epidemiology and Preventive Medicine, School of Public Health, School of Medicine, Tel Aviv University, Tel Aviv 6997801, Israel; 7 Faculty of Medicine, University of Nicosia, 2408 Nicosia, Cyprus; 8 Biostatistics Research Unit, University Health Network, Toronto, ON M5G 2C4, Canada; ella.huszti@thebru.ca; 9 Sheba Lung Transplant Program, Institute of Pulmonary Medicine, Sheba Medical Center, Tel-Hashomer, Ramat Gan 5266202, Israel

**Keywords:** pneumonia, mortality, bronchoalveolar lavage, immunocompetent, propensity score matching, respiratory infections, diagnostic procedures

## Abstract

**Background:** Bronchoalveolar lavage (BAL) is frequently employed for diagnostic purposes in immunocompromised patients with pneumonia, yet its role in immunocompetent individuals remains debated. The study investigates whether BAL is associated with reduced mortality in immunocompetent patients hospitalized with pneumonia. **Methods:** A retrospective cohort study was conducted at SMC, including 13,180 immunocompetent patients hospitalized with pneumonia from 2007 to 2024. Patients who underwent BAL (n = 96) were matched 1: 4 to a control group (n = 384) using Propensity score matching based on age, gender, severity scores and comorbidities. Mortality was assessed at 30, 60, and 90 days using logistic regression models, adjusting for length of stay, inflammatory markers, albumin levels, smoking status, and BMI. **Results:** In the matched cohort, 30-day mortality did not differ significantly between groups (20.8% in BAL vs. 19.5% in controls, *p* = 0.886), and no significant association was observed after adjustment (OR = 0.95 [0.51–1.73]. However, both 60-day and 90-day mortality were significantly higher in the BAL group (39.6% vs. 24.7% and 45.8% vs. 26.8%, respectively; *p* < 0.01 for both), and these differences persisted after adjustment (60 days: OR = 1.74 [1.03–2.94]; 90 days: OR = 2.12 [1.27–3.54]). **Conclusions:** In this cohort of immunocompetent patients hospitalized with pneumonia, BAL was not associated with short-term survival. Clinicians should carefully weigh the potential risks and benefits of BAL in this population. Further studies are needed to identify patient subgroups that might benefit from BAL through enhanced diagnostic or therapeutic approaches.

## 1. Introduction

Pneumonia is a major contributor to global morbidity and mortality, and it remains the leading cause of death from infectious diseases worldwide [1,2,3]. The onset of pneumonia is influenced by a combination of factors, including host susceptibility, pathogen virulence, and the inoculum of microorganisms. Identifying pathogens may be required in some cases for guiding antibiotic therapy, reducing antimicrobial resistance, and lowering healthcare-associated costs [4,5].

Bronchoalveolar lavage (BAL) is a widely used diagnostic tool in immunocompromised individuals to investigate pulmonary infiltrates, given their higher susceptibility to opportunistic infections and the potential for poor outcomes associated with delayed pathogen identification [6,7,8,9,10,11]. In these cases, BAL has demonstrated high diagnostic accuracy, particularly when combined with advanced diagnostic techniques such as polymerase chain reaction (PCR) and antigen detection assays, enabling precise identification of pathogens and optimized antimicrobial therapy [12,13,14,15]. However, the role of BAL in immunocompetent patients with pneumonia has not been established. While BAL may facilitate pathogen identification, its diagnostic yield and impact on clinical outcomes are less certain in immunocompetent patients.

Given the limited evidence, it is essential to explore whether BAL can contribute to improved clinical outcomes, and in particular reduced mortality, in immunocompetent patients hospitalized with pneumonia. Specifically, precise pathogen identification facilitated by BAL could guide targeted antimicrobial therapy, potentially reducing treatment failure and toxicity. Understanding the utility of BAL in this context is crucial for guiding clinical decision-making and treatment strategies. This study aimed to assess whether BAL confers a survival benefit in immunocompetent patients hospitalized with pneumonia.

## 2. Materials and Methods

### 2.1. Study Design and Population

We conducted an observational, single-center, retrospective cohort study of patients hospitalized with pneumonia between 12 October 2007 and 29 August 2024. The study adhered to the principles of the Declaration of Helsinki and received institutional review board approval. Data were retrieved using MDClone’s ADAMS platform, a self-service query tool that provides comprehensive patient-level data of wide-ranging variables in a defined time frame around an index event (https://mdclone.com/). We included all patients admitted to the internal medicine department with clinician-diagnosed pneumonia, classified according to the International Classification of Diseases, 10th Revision (ICD-10). Data was extracted from electronic medical records and censored on 5 September 2024. Immunocompromised patients were defined based on modified Infectious Diseases Society of America (IDSA) 2013 guidelines [16], including combined immunodeficiency disorders, recent chemotherapy (within 12 months), solid organ transplantation, HIV-positive status, corticosteroid therapy at doses of 15 mg/day or more of prednisone or equivalent, use of biologic immune modulators, active hematologic malignancies, myeloproliferative disorders, or use of steroid-sparing immunosuppressants [16]. Immunocompromised patients and patients lacking critical demographic or hospitalization data, such as age or length of hospitalization, were excluded. We also excluded patients diagnosed with hospital-acquired pneumonia (HAP) or ventilator-associated pneumonia (VAP), as these subtypes were not the focus of this study. We have reviewed and manually curated the electronic medical records of all patients in the BAL group to ensure data quality. The curation encompassed all medical documentation from their hospitalization, including and pulmonologists’ consultations, to confirm that the patients were immunocompetent, hospitalized in an internal medicine ward, and that the BAL test was performed to identify the causative pathogen of pneumonia. Patients who had BAL performed in the intensive care unit (ICU) before admission to the internal medicine ward or those who underwent BAL more than 32 days after department admission were also excluded. To further refine our cohort and eliminate potential confounders, we excluded patients with blood counts highly suggestive of hematologic malignancy, defined by thresholds of white blood cell (WBC) count exceeding 100 × 10^9^/L, neutrophil count exceeding 70 × 10^9^/L, lymphocyte count exceeding 50 × 10^9^/L, or platelet count exceeding 1000 × 10^9^/L.

### 2.2. Statistical Analysis

We collected data necessary for calculating the Pneumonia Severity Index (PSI) and CURB-65 scores, including age, comorbidities, vital signs, and laboratory values, to enable their use in matching analyses [17,18]. To ensure accurate scoring and analyses, we applied specific exclusion criteria for missing data. Due to substantial missing data in the following key clinical variables—respiratory rate (available for 48% of patients), hematocrit levels (66%), and chest X-ray findings (61%)—we excluded patients missing any two of these variables, as missing multiple parameters could bias results. Since partial pressure of oxygen (PaO_2_) data were available for only 5% of the patients, we did not include PaO_2_ in PSI calculations. For the remaining missing data, most variables had less than 20% missing data. We employed data imputation techniques to address these missing values. For continuous variables, we used median imputation by substituting missing values with the median of the available data. For categorical variables, we applied mode imputation by replacing missing entries with the most frequently occurring value.

Demographic characteristics were presented as counts and percentages for categorical variables and median (IQR) for continuous variables. To balance the BAL and control groups, 1:4 propensity score matching (PSM) was employed using the R MatchIt package (default parameters: method—“nearest”, distance—“glm”, link—“logic”) [19]. Covariates included in the PSM model were age, gender, length of hospitalization, PSI score (excluding PaO_2_), CURB-65 score, and comorbidities such as pulmonary diseases, cardiovascular diseases, congestive heart failure, diabetes, hypertension, and chronic kidney disease. Associations between BAL and all-cause mortality were assessed through logistic regression, and a multivariable analysis was conducted with adjustment for body mass index (BMI), smoking status, length of stay (LOS), C-reactive protein (CRP), white blood cell (WBC) count, and albumin levels. These variables were not included in the PSM model but were further adjusted to control for residual confounding. An exception was LOS, which was included in both the bivariable and multivariable analysis despite being used in the matching process, as notable differences in LOS persisted between the groups after PSM. Finally, to verify the consistency of our results, we performed a sensitivity analysis using a multivariable logistic regression model on the entire unmatched cohort (n = 13,180). This model was adjusted for all covariates used in the propensity score matching (age, sex, comorbidities, PSI, CURB-65) as well as BMI, smoking status, length of stay, CRP, WBC count, and albumin levels. All statistical analysis was conducted using R software (version 4.0.3), with a two-sided *p*-value < 0.05 considered statistically significant.

## 3. Results

### 3.1. Study Population

**Figure 1:**
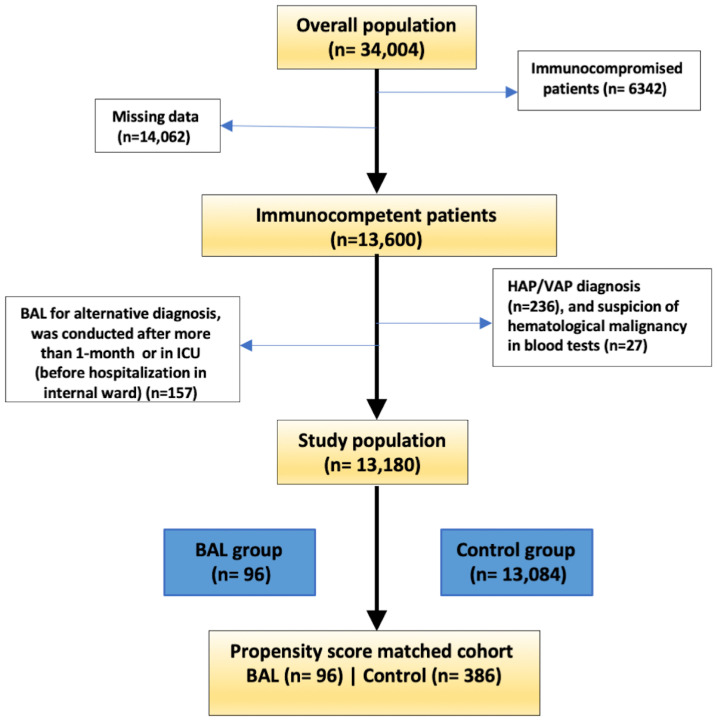
CONSORT Diagram Depicting Patient Flow Through the Study. The diagram illustrates the flow of patients through the study, including initial screening, exclusions, and final cohort selection.

**Figure 2:**
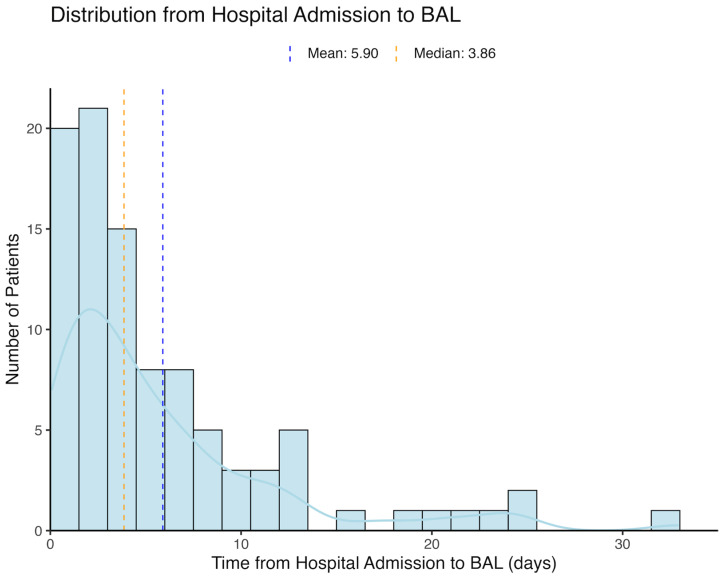
Timing of BAL Procedure. This histogram illustrates the distribution of time (in days) from hospital admission to the bronchoalveolar lavage (BAL) procedure. The blue line represents the estimated kernel density function (density curve) of the distribution.

Between 2007 and 2024, a total of 34,004 patients were admitted to the internal medicine department with a diagnosis of pneumonia. After excluding immunocompromised patients (n = 6342) and those with missing data (n = 14,062), the cohort included 13,600 patients, of whom 275 underwent BAL (Figure 1). We further excluded patients with HAP or VAP (n = 236), those with blood tests highly suggestive of hematological malignancy (n = 27), those who had an alternative indication for BAL, and those who underwent BAL in the intensive care unit prior to admission to the internal medicine ward or received BAL more than 32 days after admission (n = 157). The final cohort included 13,180 patients, of whom 96 underwent BAL, with a median time from department admission to BAL of 3.86 days (Figure 2).

The BAL group included 65% males with a median age of 70 years [61, 77], younger than the median age of patients in the control group (81 years [70, 88], 52% male, Table 1). The BAL group also exhibited significantly higher C-reactive protein levels compared to the control group (111 [63, 218] vs. 91 [33, 164] mg/L, *p* < 0.01), although there was no significant difference in the WBC count (12.2 [9.1, 15.6] vs. 11.5 [8.5, 15.5] × 10^9^/L, *p* = 0.3). Other laboratory values that differed significantly between the BAL and control groups included creatinine (0.93 [0.7, 1.3] vs. 1.1 [0.8, 1.5] mg/dL, *p* < 0.05), urea (38.5 [30, 67.5] vs. 52 [36, 79] mg/dL, *p* < 0.001), and albumin (3.2 [2.8, 3.4] vs. 3.4 [3.1, 3.8] g/dL, *p* < 0.001). Comorbidity patterns showed several notable differences between groups. Hypertension and chronic kidney disease were markedly less common in BAL patients than in controls (28% vs. 51%, *p* < 0.001 and 4% vs. 12%, *p* = 0.012, respectively). The length of stay (LOS) in the internal medicine department was significantly longer for the BAL group (median 9.9 [4.4, 14.9] days) compared to 2.9 [1.5, 6] days in the control group (*p* < 0.001). The pneumonia severity index was slightly lower in the BAL group and reached statistical significance (median 101 [76–123] vs. 106 [84–131], *p* = 0.045), whereas the CURB-65 score showed no significant difference (1 [1, 2] vs. 2 [1, 2], *p* = 0.176).

### 3.2. Propensity Score Matching and Mortality Analysis

**Figure 3:**
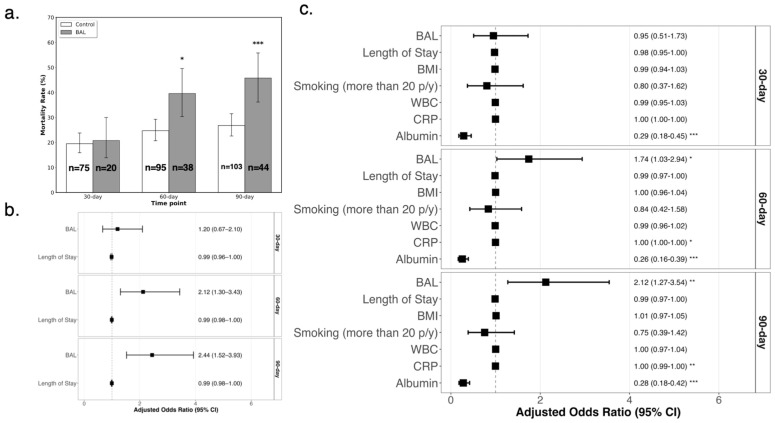
Forest Plot of Odds Ratios for Factors Associated with 30-, 60-, and 90-Day Mortality. This forest plot demonstrates the odd ratio (ORs) and 95% confidence intervals (CIs) derived from a multivariable logistic regression model assessing the impact of BAL and other clinical variables on mortality at 30, 60, and 90 days: (**a**) Crude mortality rates. (**b**) Adjusted ORs and 95% CIs for the association of BAL with 30-, 60-, and 90-day mortality, accounting for confounders. (**c**) Forest plot presenting ORs and 95% CIs from a multivariable logistic regression model. *: *p* < 0.05; **: *p* < 0.01; ***: *p* < 0.001.

Following propensity score matching, a balanced cohort of 480 patients was created, comprising 96 in the BAL group and 384 in the control group (Table 2). Baseline characteristics, including age, gender, comorbidities, and CURB-65 and PSI scores, were well balanced between the two groups (*p* > 0.05). However, the length of stay remained significantly higher in the BAL group (median 9.9 [4.4–14.9] days) compared to the control group (median 3.4 [1.6–7.4] days, *p* < 0.001). This residual imbalance was accounted for in subsequent analyses (Figure 3b,c). The BAL test for evaluating pneumonia in immunocompetent patients did not reduce 30-day all-cause mortality (20.8% vs. 19.5%, *p* = 0.78; BAL: 20/96 vs. control: 75/384). A sensitivity analysis performed on the entire unmatched population yielded consistent results, showing no significant association between BAL and 30-day mortality (adjusted OR = 1.42, 95% CI 0.80–2.41, *p* = 0.212). However, mortality was significantly higher in the BAL group at 60 days (39.6%vs. 24.7%, *p* < 0.01; BAL: 38/96 vs. control: 95/384) and 90 days (45.8% vs. 26.8%, *p* < 0.001; BAL: 44/96 vs. control: 103/384) (Figure 3a). After adjusting for LOS, the 30-day mortality difference remained non-significant between groups (OR = 1.2 [0.67–2.10]), with no significant effect of LOS on mortality (OR = 0.99, [0.96–1.00]) (Figure 3b). In contrast, the 60-day and 90-day mortality differences remained significant after adjustment (OR = 2.12 [1.30–3.43]; OR = 2.44 [1.52–3.93], respectively). Mortality was similar between the BAL and control groups at 30 days (OR = 0.95 [0.51–1.73] but was significantly higher in the BAL group at 60 days (OR = 1.74 [1.03–2.94]) and 90 days (OR = 2.12 [1.27–3.54]), based on adjusted analyses (Figure 3c). Significant differences were also observed in albumin levels (30-day: OR = 0.29 [0.18–0.45]; 60-day: OR = 0.26 [0.16–0.39]; 90-day: OR = 0.28 [0.18–0.42]). ICU transfer during hospitalization occurred more frequently in the BAL group than in controls (21.9% vs. 2.3%; *p* < 0.001).

### 3.3. BAL-Related Complications

**Figure 4:**
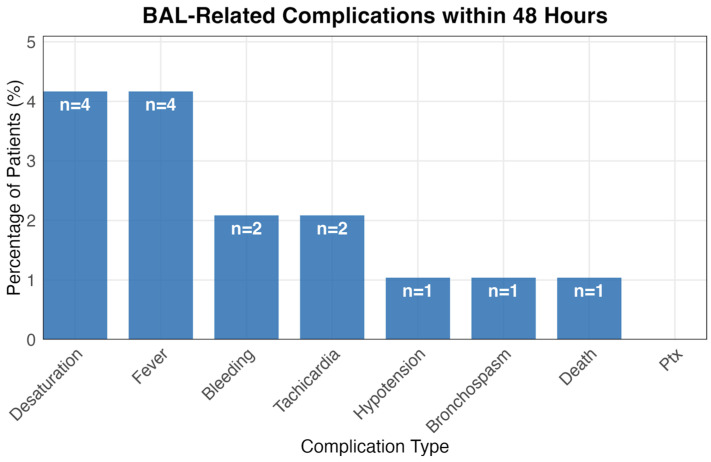
BAL-Related Complications within 48 Hours. Bar chart showing the percentage and absolute number of patients experiencing various complications within 48 h of BAL procedure.

Within 48 h of the procedure, 15.6% (15/96) of BAL patients experienced at least one complication (Figure 4). Desaturation and fever were most common (4.2% each), followed by bleeding and tachycardia (2.1% each). Hypotension, bronchospasm, and death each occurred in one patient (1.0%).

## 4. Discussion

The role of BAL in immunocompetent patients with pneumonia remains uncertain, particularly given the well-established impact of pneumonia on long-term mortality [20,21,22]. In the current study, we evaluated whether BAL is associated with reduced mortality in immunocompetent patients hospitalized with pneumonia.

Our findings suggest that BAL does not confer a short-term survival benefit in this population, as indicated by the absence of significant improvement in 30-day mortality outcomes. While BAL is primarily a diagnostic procedure rather than a therapeutic intervention, its clinical justification in this context rests on the premise that accurate diagnosis leads to better management and improved outcomes. The lack of survival benefit implies that the diagnostic yield did not translate into a tangible prognostic advantage in this cohort. These results persisted even after adjusting for potential confounders including LOS.

Nonetheless, we observed significantly higher 60-day and 90-day mortality in the BAL group. We do not interpret this as evidence of a direct causal relationship but rather as a reflection of underlying differences in disease severity. The higher rate of ICU transfers in the BAL group (21.9% vs. 2.3%, *p* < 0.001) suggesting that clinical deterioration may have prompted the use of BAL and likely contributed to the elevated 60-day and 90-day mortality. We interpret the elevated 60-day and 90-day mortality not as a direct consequence of the procedure, but rather as a reflection of residual confounding by indication. Clinicians likely selected patients for BAL who exhibited a more complex clinical course or ‘failure to thrive’ that was not fully captured by baseline severity scores (PSI/CURB-65) calculated at admission.

While the rate of ICU transfers in the BAL group aligns with previous literature, the notably lower rate in the control group suggests a disparity in disease severity during the hospitalization, despite propensity-score matching based on admission-day data [23]. Furthermore, the mortality rates observed in the BAL group exceeded those typically reported for patients hospitalized with pneumonia but were consistent with rates reported for ICU admissions with pneumonia [23,24,25]. This finding, coupled with the statistically lower albumin levels in the BAL group (30-day: OR = 0.29 [0.18–0.45]; 60-day: OR = 0.26 [0.16–0.39]; 90-day: OR = 0.28 [0.18–0.42]), suggests that these patients were frailer overall. Albumin is a well-recognized prognostic marker for mortality in hospitalized patients (Figure 3b) [26,27].

Another noteworthy finding is that a longer LOS did not appear to increase mortality in our matched cohort. While some studies report a similar lack of correlation in pneumonia, others have demonstrated the opposite [24,28,29]. LOS is influenced by various clinical factors, including PSI, comorbidities, and complications [17,30,31,32]. In our study, despite failing to match LOS (median hospitalization of 9.9 vs. 3.4 days in the BAL and control groups, respectively; *p* < 0.001), we successfully match on PSI and other major comorbidities. Consequently, any additional effect of LOS on mortality may have been attenuated in the matched group. In the unmatched population, a longer LOS was significantly associated with higher mortality at 60 and 90 days. By contrast, in the matched cohort, after accounting for confounders, LOS was no longer independently associated with mortality outcomes. (Figure 3b).

Our study is among the first to specifically highlight the absence of a clear mortality benefit from BAL in immunocompetent patients with pneumonia. Notably, we excluded patients with VAP and HAP since these subgroups were not the focus of our investigation. Evidence strongly support BAL’s utility in immunocompromised patients, where timely and accurate diagnosis is critical, and delays are associated with high mortality rates [6,7,8,9,10,11]. On average, 51.1% of immunocompromised patients with pulmonary infiltrates undergoing BAL receive a confirmed diagnosis, a figure further improved by advanced diagnostic assays [12,13,14,15].

In patients with HAP or VAP, international guidelines recommend obtaining distal respiratory samples, with BAL often providing comprehensive diagnostic data [4,33]. However, meta-analyses indicate that BAL’s diagnostic yield in VAP can be relatively low in critically ill patients. Moreover, specific assays, even those with a 98% negative predictive value, did not consistently reduce the use of empirical treatment rates for VAP [34,35]. Historically, BAL was also considered valuable for diagnosing non-resolving pneumonia in immunocompetent patients [36]. However, with the advent of numerous non-invasive laboratory tests for pathogen detection and the availability of broad-spectrum antibiotics, the role of BAL in this population is increasingly uncertain.

Certain clinical contexts still warrant the use of BAL, such as cases with moderate to high suspicion of fungal pneumonia based on host factors, epidemiology, radiographic findings, or a diagnosis of non-resolving pneumonia, particularly when non-invasive tests yield negative results [13]. Additional indications include suspected mycobacterium tuberculosis infection or severe viral pneumonia with a potential fungal co-infection [15,37,38,39]. However, our study focused on mortality rather than diagnostic yield. The findings suggest that in immunocompetent patients hospitalized with pneumonia, BAL does not confer a significant survival advantage. Considering the procedure’s inherent risks—including worsening hypoxemia, bleeding, pneumothorax, infection, arrhythmias, seizures, cardiac arrest and other complications—clinicians should carefully balance these risks against the potential diagnostic or therapeutic benefits before proceeding [6,40,41,42]. In our cohort, BAL-related complications within 48 h occurred in 15.6% of patients (15/96), with desaturation and fever being the most common adverse events (4.2% each), followed by bleeding and tachycardia (2.1% each) (Figure 4).

While our study provides valuable insights into the role of BAL in immunocompetent patients with pneumonia, it is important to consider both its strengths and limitations. Notable strengths include comprehensive data collection and the application of two complementary methods: propensity score matching (PSM) and multivariable regression analysis, to mitigate residual confounding, a potential limitation inherent to retrospective single-center studies. PSM helped balance observed baseline characteristics between groups, while multivariable regression further controlled for additional covariates, addressing confounding factors that could influence mortality outcomes. However, several limitations must be acknowledged. Indication bias may have influenced our findings, as the decision to perform BAL was based on the pulmonologist’s clinical judgment. Additionally, selection bias due to the exclusion of a substantial number of patients with missing data may limit the generalizability of our results. The relatively small size of the BAL group further restricts the scope of analysis and reduces statistical power. Finally, our primary focus on mortality as the outcome measure may not fully capture the broader clinical benefits or risks associated with BAL, such as its impact on diagnostic accuracy, treatment decisions, or long-term patient outcomes. Future research should prioritize prospective, multicenter trials with predefined criteria for BAL use in immunocompetent patients. Identifying specific subgroups most likely to benefit from BAL is essential, as is the evaluation of additional outcomes beyond mortality, such as ICU admission rates, readmission rates, and quality of life- areas that were beyond the scope of this study. Finally, the development of innovative diagnostic tools and biomarkers may enable better patient stratification and guide a more targeted, efficient approach to utilizing BAL in immunocompetent patients with pneumonia.

## 5. Conclusions

In conclusion, BAL did not demonstrate a mortality benefit in immunocompetent patients hospitalized with pneumonia. These findings highlight the need for clinicians to exercise caution when considering BAL in this population, particularly given its associated risks and the availability of noninvasive diagnostic alternatives. While BAL remains an indispensable diagnostic tool in specific contexts, such as immunocompromised individuals, further research is needed to explore whether BAL may be associated with higher mortality over extended follow-up periods. Additionally, studies should aim to identify the precise clinical scenarios in which BAL could provide meaningful benefits for immunocompetent patients.

## Figures and Tables

**Table 1:** Baseline Demographic and Clinical Characteristic of the Study Cohort. This table summarizes the baseline demographic and clinical characteristics of the study cohort, including comparisons between the BAL and control groups.

Characteristic	Control (n = 13,084)	BAL (n = 96)	*p*-Value
**Demographics**			
Age—yr	81.0 (70.1–88.2)	69.9 (61.1–77.1)	<0.001
Male sex—no. (%)	6865 (52.5)	62 (64.6)	0.023
Nursing home resident—no. (%)	790 (6.0)	5 (5.2)	0.900
**Habits**			
Smoking > 20 pack-years—no. (%)	1123 (8.6)	21 (21.9)	<0.001
Body-mass index ‡	25.7 (24.4–27.2)	25.7 (23.1–25.7)	0.006
**Vital Signs**			
Respiratory rate—breaths/min	20.0 (18.0–24.0)	20.0 (18.0–21.2)	0.662
Hypotension §—no. (%)	5866 (44.8)	45 (46.9)	0.766
Temperature abnormality ¶—no. (%)	3638 (27.8)	19 (19.8)	0.103
Heart rate—beats/min	92.0 (78.0–107.0)	95.5 (81.8–110.0)	0.203
Altered mental status—no. (%)	670 (5.1)	10 (10.4)	0.035
Mechanical ventilation—no. (%)	715 (5.5)	14 (14.6)	<0.001
**Laboratory Values**			
White-cell count—×10^9^/L	11.5 (8.5–15.5)	12.2 (9.1–15.6)	0.394
Neutrophils—×10^9^/L	9.3 (6.5–13.0)	9.9 (7.1–13.4)	0.365
Lymphocytes—×10^9^/L	1.0 (0.7–1.6)	1.0 (0.6–1.7)	0.887
Hemoglobin—g/dL	12.0 (11.4–12.7)	12.0 (12.0–12.1)	0.740
Hematocrit—%	38.0 (34.5–42.0)	38.0 (38.0–43.9)	0.042
Platelet count—×10^9^/L	231.0 (176.0–301.0)	255.0 (199.5–399.2)	0.002
Sodium—mmol/L	137.0 (134.0–140.2)	136.0 (133.5–138.6)	0.007
Glucose—mg/dL	141.0 (116.0–188.0)	131.0 (108.8–176.2)	0.060
Urea—mg/dL	52.0 (36.0–79.0)	38.5 (30.0–67.5)	<0.001
Albumin—g/dL	3.4 (3.1–3.8)	3.2 (2.8–3.4)	<0.001
Creatinine—mg/dL	1.1 (0.8–1.5)	0.9 (0.7–1.3)	0.005
Total bilirubin—mg/dL	0.6 (0.4–0.8)	0.7 (0.5–0.9)	0.127
Lactate dehydrogenase—U/L	279.0 (222.0–365.0)	279.0 (219.0–437.0)	0.651
C-reactive protein—mg/L	91.1 (33.5–163.6)	111.3 (63.1–218.2)	0.003
pH	7.4 (7.3–7.4)	7.4 (7.3–7.4)	0.056
Lactate—mmol/L	18.0 (14.0–25.0)	18.0 (15.0–26.0)	0.208
**Chest Radiographic Finding**			
Pleural effusion—no. (%)	346 (2.6)	3 (3.1)	0.743
**Coexisting Conditions—no. (%)**			
COPD	1483 (11.3)	11 (11.5)	1.000
Bronchiectasis	138 (1.1)	2 (2.1)	0.272
Interstitial lung disease	60 (0.5)	0 (0.0)	1.000
Ischemic heart disease	2429 (18.6)	10 (10.4)	0.055
Peripheral vascular disease	627 (4.8)	5 (5.2)	1.000
Previous stroke or TIA	2378 (18.2)	10 (10.4)	0.067
Liver disease	191 (1.5)	2 (2.1)	0.653
Congestive heart failure	1898 (14.5)	8 (8.3)	0.117
Diabetes	3756 (28.7)	20 (20.8)	0.113
Hypertension	6673 (51.0)	27 (28.1)	<0.001
Chronic kidney disease	1599 (12.2)	4 (4.2)	0.012
**Severity and Length of Stay**			
CURB-65 score ‖	2.0 (1.0–2.0)	1.0 (1.0–2.0)	0.176
Pneumonia severity index	106.0 (84.0–131.0)	100.5 (76.0–123.0)	0.045
Length of stay—days	2.9 (1.5–6.0)	9.9 (4.4–14.9)	<0.001

‡ Body-mass index is the weight in kilograms divided by the square of the height in meters. § Hypotension is defined as systolic blood pressure <90 mmHg. ¶ Temperature abnormality is defined as temperature <36 °C or >38 °C. ‖ CURB-65 scores range from 0 to 5, with higher scores indicating more severe illness.

**Table 2:** Comparison of Baseline Characteristics Before and After Propensity Score Matching.

	Before Matching			After Matching		
Characteristics	BAL	Control	*p*-Value	BAL	Control	*p*-Value
n	96	13,084		96	384	
Age (years)	69.92 [61.11, 77.13]	80.97 [70.13, 88.15]	<0.001	69.92 [61.11, 77.13]	70.91 [54.02, 81.73]	0.563
Pneumonia Severity Index	100.50 [76.00, 123.00]	106.00 [84.00, 131.00]	0.045	100.50 [76.00, 123.00]	99.00 [76.00, 128.00]	0.803
CURB-65	1.00 [1.00, 2.00]	2.00 [1.00, 2.00]	0.176	1.00 [1.00, 2.00]	1.00 [1.00, 2.00]	0.752
Length of Stay (days)	9.92 [4.36, 14.91]	2.88 [1.49, 5.96]	<0.001	9.92 [4.36, 14.91]	3.40 [1.62, 7.37]	<0.001
Gender	62 (64.6)	6865 (52.5)	0.018	62 (64.6)	263 (68.5)	0.464
Pulmonary Diseases	13 (13.5)	1619 (12.4)	0.729	13 (13.5)	61 (15.9)	0.569
Cardiovascular diseases	22 (22.9)	4360 (33.3)	0.031	22 (22.9)	86 (22.4)	0.913
Congestive Heart Failure	8 (8.3)	1898 (14.5)	0.087	8 (8.3)	28 (7.3)	0.729
Chronic Kidney Disease	4 (4.2)	1599 (12.2)	0.016	4 (4.2)	12 (3.1)	0.611
Diabetes	20 (20.8)	3756 (28.7)	0.089	20 (20.8)	78 (20.3)	0.910
Hypertension	27 (28.1)	6673 (51.0)	<0.001	27 (28.1)	100 (26.0)	0.679

BAL denotes bronchoalveolar lavage. CURB-65 denotes confusion, urea ≥ 7 mmol/L, respiratory rate ≥ 30/min, blood pressure (systolic < 90 mm Hg or diastolic < 60 mm Hg), and age ≥ 65 years.

## Data Availability

The data presented in this study are available on request from the corresponding author. The data are not publicly available due to privacy and ethical restrictions.

## Abbreviations

The following abbreviations are used in this manuscript:

BAL	Bronchoalveolar Lavage
BMI	Body Mass Index
CRP	C-Reactive Protein
CURB-65	Confusion, Urea, Respiratory rate, Blood pressure, Age ≥65
HAP	Hospital-Acquired Pneumonia
ICD-10	International Classification of Diseases, 10th Revision
ICU	Intensive Care Unit
IDSA	Infectious Diseases Society of America
IQR	Interquartile Range
LOS	Length of Stay
OR	Odds Ratio
PCR	Polymerase Chain Reaction
PSI	Pneumonia Severity Index
PSM	Propensity Score Matching
VAP	Ventilator-Associated Pneumonia.

## References

[1] Vos T., Lim S.S., Cristiana A., Kalankesh L.R., Zimsen S.R.M., Naghavi M., Murray C.J.L., Cederroth C.R., Samy A., Silva J.P. (2020). Global burden of 369 diseases and injuries in 204 countries and territories, 1990–2019: A systematic analysis for the Global Burden of Disease Study 2019. Lancet.

[2] Ramirez J.A., Wiemken T.L., Peyrani P., Arnold F.W., Kelley R., Mattingly W.A., Nakamatsu R., Pena S., Guinn B.E., Furmanek S.P. (2017). Adults Hospitalized With Pneumonia in the United States: Incidence, Epidemiology, and Mortality. Clin. Infect. Dis..

[3] Murphy S.L., Kochanek K.D., Xu J., Arias E. (2021). Mortality in the United States, 2020. NCHS Data Brief.

[4] Torres A., Cilloniz C., Niederman M.S., Menéndez R., Chalmers J.D., Wunderink R.G., van der Poll T. (2021). Pneumonia. Nat. Rev. Dis. Primers.

[5] File T.M., Ramirez J.A. (2023). Community-Acquired Pneumonia. N. Engl. J. Med..

[6] Gilbert C.R., Lerner A., Baram M., Awsare B.K. (2013). Utility of flexible bronchoscopy in the evaluation of pulmonary infiltrates in the hematopoietic stem cell transplant population—A single center fourteen year experience. Arch. Bronconeumol..

[7] Shannon V.R., Andersson B.S., Lei X., Champlin R.E., Kontoyiannis D.P. (2010). Utility of early versus late fiberoptic bronchoscopy in the evaluation of new pulmonary infiltrates following hematopoietic stem cell transplantation. Bone Marrow Transplant..

[8] Rañó A., Agustí C., Benito N., Rovira M., Angrill J., Pumarola T., Torres A. (2002). Prognostic Factors of Non-HIV Immunocompromised Patients With Pulmonary Infiltrates. Chest.

[9] Rano A. (2001). Pulmonary infiltrates in non-HIV immunocompromised patients: A diagnostic approach using non-invasive and bronchoscopic procedures. Thorax.

[10] Al-Qadi M.O., Cartin-Ceba R., Kashyap R., Kaur S., Peters S.G. (2018). The Diagnostic Yield, Safety, and Impact of Flexible Bronchoscopy in Non-HIV Immunocompromised Critically Ill Patients in the Intensive Care Unit. Lung.

[11] Brownback K., Simpson S. (2013). Association of bronchoalveolar lavage yield with chest computed tomography findings and symptoms in immunocompromised patients. Ann. Thorac. Med..

[12] Choo R., Anantham D. (2019). Role of bronchoalveolar lavage in the management of immunocompromised patients with pulmonary infiltrates. Ann. Transl. Med..

[13] Azar M.M. (2024). A Diagnostic Approach to Fungal Pneumonia. Chest.

[14] Oren I., Hardak E., Zuckerman T., Geffen Y., Hoffman R., Yigla M., Avivi I. (2016). Does molecular analysis increase the efficacy of bronchoalveolar lavage in the diagnosis and management of respiratory infections in hemato-oncological patients?. Int. J. Infect. Dis..

[15] Theron G., Peter J., Meldau R., Khalfey H., Gina P., Matinyena B., Lenders L., Calligaro G., Allwood B., Symons G. (2013). Accuracy and impact of Xpert MTB/RIF for the diagnosis of smear-negative or sputum-scarce tuberculosis using bronchoalveolar lavage fluid. Thorax.

[16] Rubin L.G., Levin M.J., Ljungman P., Davies E.G., Avery R., Tomblyn M., Bousvaros A., Dhanireddy S., Sung L., Keyserling H. (2014). Executive Summary: 2013 IDSA Clinical Practice Guideline for Vaccination of the Immunocompromised Host. Clin. Infect. Dis..

[17] Fine M.J., Auble T.E., Yealy D.M., Hanusa B.H., Weissfeld L.A., Singer D.E., Coley C.M., Marrie T.J., Kapoor W.N. (1997). A Prediction Rule to Identify Low-Risk Patients with Community-Acquired Pneumonia. N. Engl. J. Med..

[18] Lim W.S. (2003). Defining community acquired pneumonia severity on presentation to hospital: An international derivation and validation study. Thorax.

[19] Rosenbaum P.R., Rubin D.B. (1983). The central role of the propensity score in observational studies for causal effects. Biometrika.

[20] Waterer G.W., Kessler L.A., Wunderink R.G. (2004). Medium-Term Survival after Hospitalization with Community-Acquired Pneumonia. Am. J. Respir. Crit. Care Med..

[21] Bruns A.H.W., Oosterheert J.J., Cucciolillo M.C., El Moussaoui R., Groenwold R.H.H., Prins J.M., Hoepelman A.I.M. (2011). Cause-specific long-term mortality rates in patients recovered from community-acquired pneumonia as compared with the general Dutch population. Clin. Microbiol. Infect..

[22] Bordon J., Wiemken T., Peyrani P., Paz M.L., Gnoni M., Cabral P., del Carmen Venero M., Ramirez J., CAPO Study Group (2010). Decrease in Long-term Survival for Hospitalized Patients With Community-Acquired Pneumonia. Chest.

[23] Jain S., Self W.H., Wunderink R.G., Fakhran S., Balk R., Bramley A.M., Reed C., Grijalva C.G., Anderson E.J., Courtney D.M. (2015). Community-Acquired Pneumonia Requiring Hospitalization among U.S. Adults. N. Engl. J. Med..

[24] Peters Z.J., Ashman J.J., Schwartzman A., DeFrances C.J. (2022). National Hospital Care Survey Demonstration Projects: Examination of Inpatient Hospitalization and Risk of Mortality Among Patients Diagnosed With Pneumonia. Natl. Health Stat. Rep..

[25] Wang J., Song Y.-L. (2019). Advances in severe community-acquired pneumonia. Chin. Med. J..

[26] Viasus D., Garcia-Vidal C., Simonetti A., Manresa F., Dorca J., Gudiol F., Carratalà J. (2013). Prognostic value of serum albumin levels in hospitalized adults with community-acquired pneumonia. J. Infect..

[27] Goldwasser P., Feldman J. (1997). Association of serum albumin and mortality risk. J. Clin. Epidemiol..

[28] Han T.S., Murray P., Robin J., Wilkinson P., Fluck D., Fry C.H. (2022). Evaluation of the association of length of stay in hospital and outcomes. Int. J. Qual. Health Care.

[29] McCormick D., Fine M.J., Coley C.M., Marrie T.J., Lave J.R., Obrosky D., Kapoor W.N., E Singer D. (1999). Variation in length of hospital stay in patients with community-acquired pneumonia: Are shorter stays associated with worse medical outcomes?. Am. J. Med..

[30] Menéndez R., Ferrando D., Vallés J.M., Martínez E., Perpiñá M. (2001). Initial risk class and length of hospital stay in community-acquired pneumonia. Eur. Respir. J..

[31] Menéndez R., Cremades M.J., Martínez-Moragón E., Soler J.J., Reyes S., Perpiñá M. (2003). Duration of length of stay in pneumonia: Influence of clinical factors and hospital type. Eur. Respir. J..

[32] Dwivedi S., Madaan R., Pokharel S., Bhattarai B., Ray A., Ghosh M. (2020). Effect of Chronic Illnesses on Length of Stay and Mortality of Community Acquired Pneumonia in a Community Hospital. Am. J. Hosp. Med..

[33] Kalil A.C., Metersky M.L., Klompas M., Muscedere J., Sweeney D.A., Palmer L.B., Napolitano L.M., P O N., Bartlett J.G., Carratalà J. (2016). Management of Adults With Hospital-acquired and Ventilator-associated Pneumonia: 2016 Clinical Practice Guidelines by the Infectious Diseases Society of America and the American Thoracic Society. Clin. Infect. Dis..

[34] Fernando S.M., Tran A., Cheng W., Klompas M., Kyeremanteng K., Mehta S., English S.W., Muscedere J., Cook D.J., Torres A. (2020). Diagnosis of ventilator-associated pneumonia in critically ill adult patients—A systematic review and meta-analysis. Intensive Care Med..

[35] Hellyer T.P., McAuley D.F., Walsh T.S., Anderson N., Morris A.C., Singh S., Dark P., I Roy A., Perkins G.D., McMullan R. (2020). Biomarker-guided antibiotic stewardship in suspected ventilator-associated pneumonia (VAPrapid2): A randomised controlled trial and process evaluation. Lancet Respir. Med..

[36] Feinsilver S.H., Fein A.M., Niederman M.S., Schultz D.E., Faegenburg D.H. (1990). Utility of Fiberoptic Bronchoscopy in Nonresolving Pneumonia. Chest.

[37] Gioia F., Walti L.N., Orchanian-Cheff A., Husain S. (2024). Risk factors for COVID-19-associated pulmonary aspergillosis: A systematic review and meta-analysis. Lancet Respir. Med..

[38] Lu L.Y., Lee H.M., Burke A., Bassi G.L., Torres A., Fraser J.F., Fanning J.P. (2024). Prevalence, Risk Factors, Clinical Features, and Outcome of Influenza-Associated Pulmonary Aspergillosis in Critically Ill Patients. Chest.

[39] Feys S., Carvalho A., Clancy C.J., Gangneux J.-P., Hoenigl M., Lagrou K., A Rijnders B.J., Seldeslachts L., Vanderbeke L., van de Veerdonk F.L. (2024). Influenza-associated and COVID-19-associated pulmonary aspergillosis in critically ill patients. Lancet Respir. Med..

[40] Miller R.J., Casal R.F., Lazarus D.R., Ost D.E., Eapen G.A. (2018). Flexible Bronchoscopy. Clin. Chest Med..

[41] Du Rand I.A., Blaikley J., Booton R., Chaudhuri N., Gupta V., Khalid S., Mandal S., Martin J., Mills J., Navani N. (2013). British Thoracic Society guideline for diagnostic flexible bronchoscopy in adults: Accredited by NICE. Thorax.

[42] Strumpf I.J., Feld M.K., Cornelius M.J., Keogh B.A., Crystal R.G. (1981). Safety of Fiberoptic Bronchoalveolar Lavage in Evaluation of Interstitial Lung Disease. Chest.

